# Celiac Disease in Children, Particularly with Accompanying Type 1 Diabetes, Is Characterized by Substantial Changes in the Blood Cytokine Balance, Which May Reflect Inflammatory Processes in the Small Intestinal Mucosa

**DOI:** 10.1155/2019/6179243

**Published:** 2019-05-12

**Authors:** Tamara Vorobjova, Aili Tagoma, Astrid Oras, Kristi Alnek, Kalle Kisand, Ija Talja, Oivi Uibo, Raivo Uibo

**Affiliations:** ^1^Department of Immunology, Institute of Biomedicine and Translational Medicine, University of Tartu, Ravila 19, 51014 Tartu, Estonia; ^2^Department of Pediatrics, Institute of Clinical Medicine, University of Tartu, Estonia; ^3^Children's Clinic, Tartu University Hospital, Tartu, Estonia

## Abstract

Cytokines play a pivotal role in the maintenance of intestinal homeostasis inducing pro- or anti-inflammatory response and mucosal barrier function in celiac disease (CD) and type 1 diabetes (T1D). We aimed to compare the levels of pro- and anti-inflammatory cytokines in CD patients without and with coexisting T1D, as well as to evaluate its association with the presence of enteroviruses (EV), regulatory T cells (Tregs), and dendritic cells (DCs) in small bowel mucosa. Altogether, 72 patients (median age 10.1 years) who had undergone small bowel biopsy were studied. The study group consisted of 24 patients with CD (median age 6.5 years), 9 patients with CD and concomitant T1D (median age 7.0 years), two patients with T1D (median age 8.5 years), and 37 patients (median age 14.0 years) with functional gastrointestinal disorders (FGD) and a normal small bowel mucosa as controls. The levels of 33 cytokines in serum were measured by multiple analysis using the Milliplex® MAP Magnetic Bead assay. The densities of FOXP3+ Tregs, CD11c+ DC, indoleamine 2,3-dioxygenase+ (IDO+) DC, langerin+ (CD207+) DCs, and EV were evaluated by immunohistochemistry as described in our previous studies. Circulating anti-EV IgA and IgG were evaluated using ELISA. The most important finding of the study is the significant increase of the serum levels of IL-5, IL-8, IL-13, IL-15, IL-17F, IL-22, IL-27, IP-10, MIP-1*β*, sIL-2R*α*, sTNFRII, and TNF*α* in CD patients compared to controls and its correlation with the degree of small bowel mucosa damage graded according to the Marsh classification. The leptin level was higher in females in all study groups. The levels of IL-2, IL-6, IL-12 (P70), IL-15, IP-10, and IFN*γ* correlated significantly with the density of FOXP3+ Tregs in *lamina propria* of the small bowel mucosa, which supports the evidence about the signaling role of these cytokines in the peripheral maintenance of FOXP3+ Tregs. At the same time, a significant negative correlation occurred between the level of IL-4 and density of FOXP3+ Tregs in controls. Another important finding of our study was the correlation of IL-17F, IP-10, sTNFRII, MCP-1, and GM-CSF with the density of EV-positive cells in the *lamina propria* of the small bowel mucosa. Correlation of MIP-1 (CCL-4) with CD103+ DC and langerin+ DC densities may point to their significance in the recruitment of immune cells into the *lamina propria* and in driving the inflammatory response in CD patients. Our results suggest the predominance of Th1 and Th17 immune responses over EV VP1 protein in CD and T1D patients. The significant elevation of Th2 cytokines, like IL-5 and IL-13, but not IL-4, in CD patients and its correlation with the degree of small bowel mucosa damage could reflect the role of these cytokines in gut defense and inflammation.

## 1. Introduction

Celiac disease (CD) and type 1 diabetes (T1D) are common autoimmune disorders in childhood. In the pathogenesis of both diseases, the importance of the gut's immune system has been demonstrated by several authors [1–4]. The altered immune response to ingested wheat gluten leads to inflammation and villous atrophy in the small bowel mucosa, resulting in an increased number of infiltrating lymphocytes in the epithelium and in the *lamina propria* [5]. In this association, the rise in autoantibodies against tissue transglutaminase has been shown to be a specific marker for CD [6]. Still, it is not fully clear whether other environmental agents are involved in the development of villous atrophy in CD. For example, the possible roles of enteroviruses and coxsackieviruses in damaging the small bowel mucosa and pancreatic *β*-cells in the pathogenesis of T1D have been discussed in several studies [7–11].

CD is a T cell-mediated autoimmune disease where activated T helper (Th) cells and macrophages release proinflammatory cytokines which are main players in induction of histological changes in the small intestinal mucosa [12, 13]. During this process, also dendritic cells (DCs) and regulatory T cells (Tregs) play a crucial role in intestinal homeostasis [14, 15].

Cytokines, produced by different subsets of intestinal lymphocytes, T cells, DCs, macrophages, and intestinal epithelial cells, play a pivotal role in the maintenance of intestinal homeostasis inducing the pro- or anti-inflammatory response and influencing immune cell migration and activation. Cytokines can also regulate mucosal barrier function [16–18].

There is evidence that CD and T1D patients have elevated levels of local and circulating proinflammatory and anti-inflammatory cytokines [10, 13, 19–23]. Particularly, the role of IL-15 in the activation of intraepithelial lymphocytes in CD patients has been shown by several authors [5, 12, 24]. The combined effect of IL-2, IL-7, and IL-15 on Treg/Teffector balance in T1D has also been reported [25].

In our previous studies, we showed the role of CD11c+ DCs, tolerogenic indoleamine 2,3-dioxygenase+ (IDO+) DCs, CD103+, and langerin (CD207) DC subsets in the state of the small bowel mucosa of patients with CD and T1D [26]. We highlighted the participation of diverse DC subsets in the pathological processes occurring in the small bowel mucosa in CD patients with and without T1D and showed the possible involvement of tolerogenic DCs in Treg development to maintain intestinal immunological tolerance in CD patients [27].

This study is an extension of our previous research and was carried out on children with CD and coexisting T1D who had been investigated at the time of CD diagnosis and who had not been treated with a gluten-free diet [27, 28]. We investigated 33 cytokines in blood of CD patients, in CD patients with coexisting T1D (CD + T1D), and in controls (persons with functional gastrointestinal disorders (FGD) and normal small bowel mucosa) to delineate cytokine patterns in these groups. We also aimed to investigate whether the production of systemic cytokines is associated with enterovirus (EV) infection, evaluated both immunohistochemically and by the elevated level of anti-EV IgA and IgG, as well as with the density of Tregs and dendritic cells (DCs) in the small intestine mucosa.

## 2. Material and Methods

### 2.1. Patients Studied

Seventy-two patients (44 females and 28 males, median age 10.1 years) who were admitted to the Children's Clinic of Tartu University Hospital and who underwent small bowel biopsy for clinically indicated reasons were studied (Table 1). The study group consisted of 24 patients with CD (median age 6.5 years), 9 patients with CD and concomitant T1D (median age 7.0 years), and two patients with T1D (median age 8.5 years). All 33 CD patients were recruited at the time of CD diagnosis and were not on a gluten-free diet. All had IgA antibodies to tissue transglutaminase (anti-tTG) as tested by the EliA procedure (Thermo Fisher Scientific GmbH). Nine of the newly diagnosed CD patients suffered from T1D as well. The two T1D patients (4- and 13-year-old boys) with a normal small bowel mucosa were eligible for intestinal biopsy due to gastrointestinal complaints and equivocal values of IgA anti-tTG (7.0 and 9.8 U/ml). All T1D patients received specific treatment with insulin.

The control group included 37 patients (median age 14.0 years) with functional gastrointestinal disorders (FGD) with a histologically normal small bowel mucosa and without IgA anti-tTG in blood, who had neither accompanying immune-mediated diseases nor diabetes.

The diagnosis of CD was established on the basis of the European Society for Pediatric Gastroenterology, Hepatology and Nutrition (ESPGHAN) criteria [29]. Two small bowel biopsy specimens were taken for morphological and immunohistochemical examinations. Blood for determination of cytokines and antibodies and for routine clinical analyses (blood glycose etc.) was taken at the same time. Morphologically, small bowel mucosa involvement was assessed according to the Marsh classification [30, 31], based on distal duodenum biopsies taken at gastroduodenoscopy. All 33 CD patients had partial or subtotal villous atrophy according to this classification: Marsh grade II in 1, grade IIIa in 10, grade IIIb in 16, and grade IIIc in 6 cases. All 39 non-CD patients had a normal small bowel mucosa (Marsh grade 0).

### 2.2. Quantification of Cytokines

Patients' sera were collected and stored at -80°C prior to analyses. Multiple freeze-thaw cycles (>2) were avoided. The number of collected sera was smaller for the summer period compared to the winter or autumn period (Table 2). Thirty-three cytokines were detected by multiplex analysis using different kits of Milliplex® MAP Magnetic Bead assays according to the manufacturer's recommendation (Millipore, Billerica, MA, USA) (Supplementary Table 1). Luminex 200™ (Luminex Corp., Austin, TX) was used for measurements, and XPONENT was used for data analysis. The detection limit varied for each cytokine and ranged from 0.02 ng/ml to 38 pg/ml. According to the manufacturer's specifications, the intra-assay and interassay coefficients of variation were <10% and <20%, respectively, for all assays.

### 2.3. Quantification of Immune Cells and EV Protein-Positive Cells

Immunohistochemical procedures were performed to count the density of marker-positive immune cells on the paraffin-embedded and cryostat specimens of the small bowel mucosa as presented earlier [26, 32]. In these studies, the monoclonal mouse anti-CD11c (NCL-L-CD11c-563 in 1 : 80 dilution, Novocastra™ Liquid), anti-IDO (Chemicon, Millipore Corporation, 1 : 50), anti-CD103 (integrin *α*E (N-19), sc-6606, Santa Cruz Biotechnology, 1 : 50), and anti-langerin (CD207) (N-14, Santa Cruz Biotechnology, 1 : 50) were used for DC detection. Anti-FOXP3 (clone 236A/E7, Abcam, Cambridge, UK) in 1 : 60 dilution was used for Treg detection. For concurrent detection of CD11c+ DCs and FOXP3+ Tregs in the small bowel mucosa, double staining was used; IDO+ DCs were detected by monostaining; staining for CD103 and separately for langerin (CD207) was done on frozen tissue.

In each of the 5 different microscopic fields (objective 40x and eyepiece 10x), immunostained cells were counted and cell densities were expressed as the mean number of positively stained cells per field. All sections were studied with the investigator being blinded to diagnosis and to immunological and clinical data.

For staining and counting of EV-positive cells on the paraffin sections of the small intestinal mucosa, monoclonal mouse anti-enterovirus clone 5-D8/1 (DakoCytomation) in 1 : 1000 dilution was used [32].

### 2.4. Evaluation of IgA and IgG Antibodies to Enterovirus (EV) Peptide Antigen in Blood

IgA antibodies to the EV peptide antigen were evaluated in the sera using the ELISA method as described by Viskari et al. [33]. Briefly, microtiter plates (Nunc Immunoplate, Nunc, Glostrup, Denmark) were coated with the synthetic enterovirus peptide (KEVPALTAVETGATC, Storkbio Ltd., Estonia) derived from an immunodominant region of the capsid protein VP1 [34] at 2.5 *μ*g/ml in a carbonate bicarbonate buffer (pH 9.6). The serum samples were diluted in 1 : 100 for detection of IgA and in 1 : 2000 for detection of IgG-type antibodies. The results were expressed in enzyme immunoassay units (EIU), which showed the relative antibody reactivity of the sample in comparison with the positive and negative reference sera in each assay. A seropositivity cutoff level of 15 EIU was considered significant.

### 2.5. Tissue Transglutaminase IgA Antibody (IgA-tTG) Immunoassay

Serum IgA-tTG was tested by a fully automated EliA™ Celikey® IgA assay (Pharmacia Diagnostics, Freiburg, Germany) according to the manufacturer's instructions. The values of IgA-tTG higher than 10 EliA U/ml were considered positive.

### 2.6. Genotyping of HLA-DR/DQ Polymorphisms

Information about HLA-DR/DQ polymorphisms, as a part of another study, was available for 10 CD patients, 6 CD + T1D patients, and one T1D patient. For HLA class II analyses, EDTA-treated blood samples were spotted onto Whatman filter paper, dried at room temperature, and stored then at 4°C before transport to the Turku University Immunogenetics laboratory. HLA-DR/DQ genotypes were analyzed as described elsewhere [35]. The HLA-DR/DQ haplotypes are presented in Supplementary Table 2.

### 2.7. Statistical Analysis

The results obtained for the different study groups are presented as median with the interquartile (IQR) range (25%-75%). Statistical calculations were performed using the GraphPad Prism 5.0 software, employing the nonparametric Mann-Whitney *U* test (for bivariate comparisons of two continuous variables with nonnormal distribution) and the Kruskal-Wallis test (for multiple comparisons of continuous variables with nonnormal distribution), employing also the *t*-test and Spearman's rank correlation tests. The *χ* ^2^ test (with Yates' correction) was used for nominal variables. Multiple regression and regression analyses were performed using the MedCalc (version 15.5.5) software. *P* < 0.05 was considered statistically significant.

### 2.8. Ethical Consideration

The study was reviewed and approved by the Ethics Review Committee for Human Research at the University of Tartu (protocol number 187/M-27) in accordance with the Helsinki Declaration of 1964.

All studied children and/or their parents gave written informed consent for participation.

## 3. Results

### 3.1. Cytokine Levels in Study Groups

**Figure 1:**
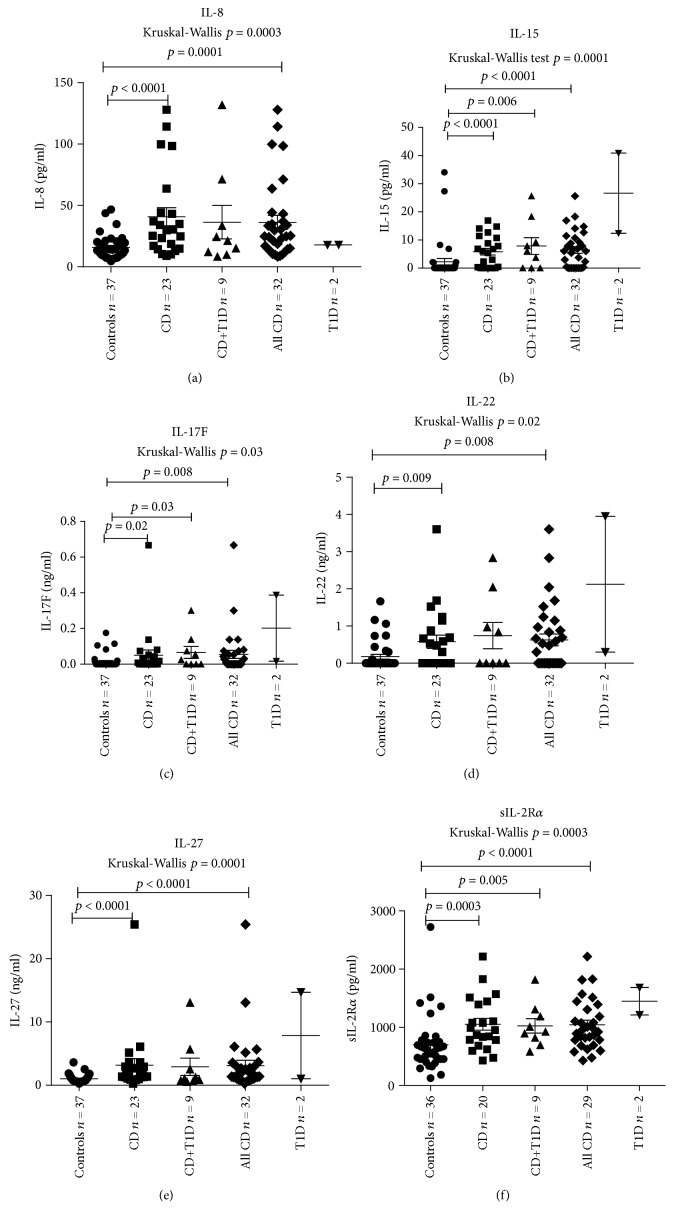
Comparison of serum levels of IL-8 (pg/ml) (a), IL-15 (pg/ml) (b), IL-17F (ng/ml) (c), IL-22 (ng/ml) (d), IL-27 (ng/ml) (e), and sIL-2R*α* (pg/ml) (f) between different patient groups (median values and interquartile range). Every dot represents the value of cytokine studied for one person. Median values between the groups were compared using the Mann-Whitney *U* test. Two T1D persons were excluded from statistical analysis. The outlier in the CD group with an IL-8 value of 207.31 pg/ml (male, 4 years old, with atrophy in small bowel mucosa Marsh grade IIIb, EV density 3+) was excluded from graphic presentation but not from statistical analysis. The outlier in the CD group with an IL-15 level of 229.89 pg/ml, with an IL-17F level of 2.46 ng/ml, with an IL-22 level of 13.46 ng/ml, and with an IL-27 level of 113.37 ng/ml (male, 9 years old, with atrophy in small bowel mucosa Marsh grade II) was excluded from graphic presentation, but not from statistical analysis. The outlier in the control group with a sIL-2Ra value of 5628.53 (male, 1 year old) was excluded from graphic presentation but not from statistical analysis.

The median level of 33 measured cytokines in different study groups is presented in Table 3. The levels of 12 of the 33 cytokines were significantly higher in CD patients compared to controls. CD patients with coexisting T1D had significantly higher levels of IL-15 (*P* = 0.006), IL-17F (*P* = 0.03), MIP-1*β* (*P* = 0.02), and sIL-2R*α* (*P* = 0.005) compared to controls. When we compared the level of the studied cytokines between CD and CD patients with coexisting T1D, no significant difference was found (*P* > 0.05 for all cytokines studied). The levels of IL-4, PAI-1, TGF*β*1, TGF*β*2, sIL-1R1, and leptin were significantly lower in CD patients compared to controls (*P* = 0.01, *P* = 0.01, *P* = 0.004, *P* = 0.006, *P* = 0.0008, and *P* = 0.03, respectively) (Supplementary Table 3). The median serum levels of IL-8, IL-15, IL-17F, IL-22, IL-27, and sIL-2R*α* in the study groups are presented in Figures 1(a)–1(f).

The levels of eight cytokines showed a highly significant correlation with the degree of small bowel mucosa damage according to the Marsh classification: IL-27 (*r* = 0.46; *P* < 0.0001), sIL-2Ra (*r* = 0.41; *P* = 0.0005), IL-15 (*r* = 0.40; *P* = 0.0005), IL-8 (*r* = 0.40; *P* = 0.0004), IL-5 (*r* = 0.30; *P* = 0.01), IP-10 (IFN*γ* inducible protein 10 or CXCL10) (*r* = 0.27; *P* = 0.02), sTNFRII (*r* = 0.26; *P* = 0.03), and MIP-1*β* (*r* = 0.25; *P* = 0.03). Additionally, the level of IP-10 was correlated with the level of IFN*γ* in CD patients (*r* = 0.40; *P* = 0.04).

The results for IgA-tTG are presented in Table 1. Among the tested cytokines, only the levels of TGF*β*1 and TGF*β*2 were correlated with levels of IgA-tTG in CD patients (*r* = 0.52; *P* = 0.008 and *r* = 0.54; *P* = 0.005, respectively). Additionally, we found that the level of TGF*β*2 was negatively correlated with level of IL-15 in all CD groups (*r* = −0.32; *P* = 0.03).

### 3.2. Seasonal Difference

The number of serum samples collected from CD patients and controls was similar for all seasons except for spring when their number was larger for control persons than for all CD patients (5 out of 17 versus 12 out of 17, *χ* ^2^ = 4.23; *P* = 0.039) (Table 2). In multiple regression analysis, only the level of IL-22 was significantly dependent on the season of blood collection (*r* partial = −0.23; *P* = 0.04) (Supplementary Table 4).

### 3.3. Cytokines and Gender

There was no significant difference in the number of male and female participants between any of the study groups (*P* > 0.05) (Table 1). In multiple regression analysis (Supplementary Table 4), the levels of the cytokines IL-1*β* (*r* partial = −0.29; *P* = 0.01), IL-2 (*r* partial = −0.24; *P* = 0.04), IL-8 (*r* partial = −0.30; *P* = 0.01), IL-12 (P70) (*r* partial = −0.28; *P* = 0.01), TGF*β*1 (*r* partial = 0.24; *P* = 0.04), sIL-1R1 (*r* partial = 0.27; *P* = 0.02), and leptin (*r* partial = −0.32; *P* = 0.006) were apparently dependent on the gender.

**Figure 2:**
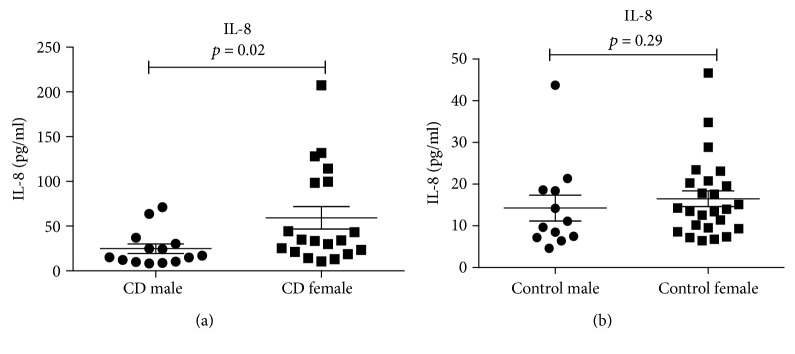
Comparison of the level of IL-8 (pg/ml) (median values and interquartile range) between males (*n* = 14) and females (*n* = 19) in CD patients using the Mann-Whitney *U* test (a). Comparison of the level of IL-8 (pg/ml) (median values) in male (*n* = 12) and female (*n* = 25) control persons using the Mann-Whitney *U* test (*P* = 0.29) (b). Every dot represents the value of IL-8 in pg/ml for one person.

Significantly higher median values of IL-8 occurred in females (34.1 pg/ml) versus males (16.0 pg/ml; *P* = 0.02) for the whole CD group (Figure 2(a)), whereas there was no statistically significant difference in IL-8 levels between males (10.4 pg/ml) and females (13.9 pg/ml; *P* = 0.29) for the control group (Figure 2(b)).

Leptin levels were higher in females in all study groups (*P* < 0.05). The level of TGF*β*1 was higher in males (median value 58782 pg/ml vs 52152 pg/ml in females, *P* = 0.04) only for the whole study group.

### 3.4. Cytokines and Age

There was no significant difference in age between males and females for any study group (*P* > 0.05); however, the median age of females in the control group was significantly higher (14.9 years) compared to the median age of females in the CD group (6.5 years) as well as in all the CD groups (7.0 years) (*P* = 0.009 and *P* = 0.004, respectively) (Table 1). Multiple regression analysis revealed that the levels of the cytokines IP-10 (*r* partial = 0.40; *P* = 0.0005), MIP-1*β* (*r* partial = −0.32; *P* = 0.006), TNF*α* (*r* partial = −0.32; *P* = 0.005), TGF*β*3 (*r* partial = −0.28; *P* = 0.01), sIL-2Ra (*r* partial = −0.27; *P* = 0.02), and leptin (*r* partial = 0.68; *P* < 0.0001) appeared to depend on age.

### 3.5. Cytokines and the Status of Small Bowel Mucosa in Relation to Markers for Tregs, DCs, and EV

The results about the density of Tregs, DCs, and EV-positive cells in the small bowel mucosa, as well as the data about anti-IgA and anti-IgG antibodies to enterovirus (EV) peptide antigen, obtained by ELISA, have been published elsewhere [26, 32] and are used as background data in the present study.

The data about statistically significant correlations between cytokines and markers for Tregs, DCs, and EV-positive cells in the small bowel mucosa of CD patients, CD patients with coexisting T1D, and the whole CD group, as well as in controls, are presented in Supplementary Table 5.

Among the significant associations found in the present study, a positive correlation between the density of FOXP3+ Tregs and the serum levels of IL-2, IL-6, IL-12 (P70), IP-10, and IFN*γ* in CD patients should be underlined. Importantly, the level of IL-15 was strongly correlated with the density of FOXP3+ Tregs in CD patients with coexisting T1D (*r* = 0.76; *P* = 0.04). The density of FOXP3+ Tregs in *lamina propria* was significantly correlated with the levels of IL-27 (*r* = 0.40; *P* = 0.002) and sIL-2Ra (*r* = 0.46; *P* = 0.0003) in the whole group of studied patients.

In control persons with a normal small bowel mucosa, no significant positive correlation was found between the density of FOXP3+ Tregs and cytokines, except for IL-4, which was negatively correlated with the density of FOXP3+ Tregs (*r* = −0.50; *P* = 0.004). It means that persons with a higher level of IL-4 had a lower density of FOXP3+ Tregs in the small bowel mucosa.

A significant correlation occurred between the density of tolerogenic IDO+ DCs and the levels of IL-10 (*r* = 0.56; *P* = 0.02), IL-12 (P70) (*r* = 0.57; *P* = 0.02), IFN*γ* (*r* = 0.53; *P* = 0.04), and GM-CSF (*r* = 0.54; *P* = 0.036) in CD patients and also between IDO+ DCs and levels of IL-27 (*r* = 0.28; *P* = 0.04) and sIL-2R*α* (*r* = 0.52; *P* ≤ 0.0001) in the whole group studied.

We found a significant correlation between the density of EV-positive cells in the small bowel mucosa and the levels of IP-10 (*r* = 0.52; *P* = 0.04) and sTNFRII (*r* = 0.45; *P* = 0.02) in CD patients, while CD patients with coexisting T1D showed a very strong correlation between the density of EV-positive cells and level of GM-CSF (*r* = 0.91; *P* = 0.006). The levels of IL-17F, IL-23, and IL-27 were significantly and strongly correlated with the level of IgG to EV in patients with CD and coexisting T1D (*r* = 0.93; *P* = 0.001, *r* = 0.73; *P* = 0.04, and *r* = 0.95; *P* = 0.001, respectively). In multiple regression analysis, the density of EV-positive cells was associated with the level of IL-8 (*r* partial = −0.45; *P* = 0.03).

The density of CD11c+ DCs was significantly correlated with the levels of IL-12 (P70), IL-17A, IL-21, and IL-23 (*r* = 0.59; *P* = 0.01, *r* = 0.56; *P* = 0.02, *r* = 0.59; *P* = 0.01, and *r* = 0.42; *P* = 0.04, respectively) in CD patients.

The density of CD103+ DCs was significantly correlated with the level of IL-7 in CD patients with coexisting T1D (*r* = 0.85; *P* = 0.01) and with the level of MIP-1*β* (*r* = 0.42; *P* = 0.03) in the whole CD group.

The density of langerin (CD207+) DCs was only correlated with the level of MIP-1*β* in CD patients (*r* = 0.52; *P* = 0.02), while in control persons with a normal bowel mucosa, the density of langerin (CD207+) DCs correlated with the levels of IL-2, IL-7, IL-12 (P70), IL-23, and TGF*β*2 (Supplementary Table 5).

## 4. Discussion

In this study, we showed that the levels of proinflammatory cytokines IL-8, IL-15, IL-17F, IL-22, TNF*α*, and sTNFRII (Th17 cytokines); anti-inflammatory cytokines IL-5, IL-13 (Th2 cytokines), IL-27, and sIL-2R*α* (Th1 cytokines); and chemokines IP-10 and MIP-1*β* in blood were significantly higher in CD patients compared to control persons with a normal small bowel mucosa. CD patients with coexisting T1D showed particularly high levels of IL-15, IL-17F, and MIP-1*β* compared to the control group. When we compared the level of the studied cytokines between CD and CD patients with coexisting T1D, no significant difference was found. The important finding of our study was that some cytokines and chemokines like IL-15, IL-8, IL-27, sIL-2R*α*, IL-5, sTNFRII, IP-10, and MIP-1*β* correlated highly significantly with the grade of small bowel mucosa damage, which plays a major role as a sign of small bowel mucosa inflammation and damage.

In our study, the level of IL-15 was significantly higher in CD patients compared to control persons and correlated significantly with the grade of small bowel mucosa damage according to the Marsh classification. Additionally, our study found a significant correlation between the level of circulating IL-15 and the density of FOXP3+ Tregs in the small bowel mucosa of CD patients with coexisting T1D, which can indicate a strong compensatory reaction of functionally inactive Tregs in these patients as IL-15 plays a pivotal role in the development of CD. IL-15 production by mononuclear cells in the *lamina propria* or by enterocytes is stimulated by gliadin [14]. According to different studies, overexpression of IL-15 in active CD may increase the number of inflammatory cells involved in the generation of epithelial damage [36, 37]. Borrelli et al. [38] showed that in patients with active CD, the level of the IL-15 cytokine and the number of IL-15+ cells in the *lamina propria* and in the epithelium of the small bowel mucosa are increased. IL-15 can mediate the activity of the anti-inflammatory pathway, including the pathways mediated by Tregs [37]. The increased level of Tregs in CD has been shown in several studies and may be induced by gliadin [28, 39]. The study of Ahmed et al. [40] demonstrated the ambiguous role of IL-15 in the control of Treg function. On the other hand, IL-15 can lead to effector cell unresponsiveness to the regulatory effect of FOXP3+ Tregs. Several studies have shown that effector T cells in the gut mucosa of patients with CD and those with CD with coexisting T1D might be insensitive to FOXP3+ Tregs because of the high level of IL-15 [14, 37]. According to Hmida et al. [41], effector T cells in patients with active CD are resistant to suppression by Tregs, which can cause loss of gluten tolerance. IL-15 was particularly increased in the serum of CD patients with T1D [40]. Also, in our study, CD patients with coexisting T1D showed a significantly higher level of IL-15 in the serum compared to control persons with a normal small bowel mucosa. Zanzi et al. [39] suggested that the activity of intestinal and peripheral Tregs might be impaired by IL-15 and the sensitivity of Tregs to IL-15 might be explained by the increased expression of IL-15 R*α* on the surface of Tregs.

The significantly increased level of circulating IL-8 found in CD patients in our study and its correlation with small bowel mucosa damage also supports the findings of several other authors [13, 42, 43]. Gliadin as a chemoattractant can directly recruit neutrophils [44]. A significant increase in IL-8 mRNA in CD patients after in vivo gluten challenge was demonstrated in the study of Brottveit et al. [45]. Hall et al. [46] showed that patients with *dermatitis herpetiformis* have elevated the serum level of IL-8 which is produced in the small bowel mucosa in response to gluten ingestion. The study of Jelínková et al. [47] established that gliadin and its fragments were able to stimulate human monocytes to significantly increase the production of IL-8 and TNF*α* which leads to damage of the intestinal mucosa in celiac patients.

In our study, according to multiple regression analysis, the level of IL-8 was associated with the density of EV-positive cells in the small bowel mucosa. Similar to our results, higher plasma levels of proinflammatory cytokines like IL-1*β*, IL-6, and IL-8 have been measured in children with enterovirus (EV71) infection [48].

The level of circulating IL-22 was significantly increased in CD patients compared to patients with a normal mucosa in our study. This can be attributed to the compensatory effect of IL-22, which has been shown in inflammatory bowel disease and ulcerative colitis [49, 50]. The role of IL-22 as a promoter of intestinal stem cell-mediated regeneration, which is critical for barrier maintenance, was established in the study of Lindeman et al. [51]. Being produced by Th17 or Th22 cells, IL-22 plays a key role in regulating mucosal barrier function and maintaining intestinal homeostasis [17, 42] as it may regulate the tight junction between intestinal epithelial cells [52].

The IL-17 family comprises several members with similar functions [53]. Among them, IL-17F is implicated in the regulation of local inflammation in the intestine [54]. We found that the level of IL-17F was significantly elevated in CD patients and in CD patients with coexisting T1D. Moreover, the elevated level of IL-17F was associated with the density of FOXP3+ Tregs and CD11c+ DCs in the small bowel mucosa when the whole group of patients was studied. Lahdenperä et al. [55] found that upregulation of intestinal FOXP3 in children with untreated CD was associated with the higher number of IL-17-positive cells or IL-17-specific mRNA levels. These authors discussed the possible association of upregulation of intestinal IL-17 immunity and protection from tissue damage in the inflamed mucosa. Mucosal IL-17A mRNA expression was elevated in CD patients also in the study of Sapone et al. [56]. These authors speculated that gliadin might directly affect the expansion of Th17 cell clones.

In this study, we found a positive correlation of the levels of IL-17F and IL-23 with the level of IgG to EV in the CD group and in the group of CD with T1D. The role of EV and particularly the role of coxsackievirus B1 (CBV1) and coxsackievirus B4 (CBV4) in the pathogenesis of T1D and CD have been shown in several studies [7, 10, 11]. CBV4 can induce production of proinflammatory cytokines which are involved in damage of insulin-producing cells [10].

Significant elevation of IL-17 and IL-23 cytokines in the sera of children infected with EV71-induced diseases has been reported in some other studies [57, 58]. Similarly, a substantial increase of the IL-17 level was found in an EV-positive group of T1D children versus control subjects and EV-negative T1D patients in the study of Abdel-Latif et al. [59]. This suggests the predominance of the Th17 immune response in T1D patients with viral infection and the role of IL-17 as a proinflammatory cytokine in the pathogenesis of the disease [60].

There is evidence that Th17 cells activated by IL-23 promote chronic tissue inflammation during infection [61] and are associated with protective immunity at the mucosa surface [62]. In our study, the level of IL-23 in CD patients was correlated not only with anti-EV IgG but also with the density of CD11c+ DCs in the *lamina propria*, which is consistent with *in vitro* studies demonstrating that CD11b+ DCs in the *lamina propria* of the small intestine preferentially induce differentiation of Th17 cells in the gut [62].

IL-27 is a member of the IL-12 family and a regulator of the Th1 response. We found significantly increased IL-27 levels in CD patients. Moreover, the level of IL-27 correlated with anti-EV IgG in CD patients with coexisting T1D and with the density of FOXP3+ Tregs and IDO+ DCs in the whole study group. Pro- and anti-inflammatory effects, including increased expression of IL-27, have previously been associated with chronic inflammatory conditions [54], which supports our finding of the correlation of the IL-27 level with small bowel mucosa damage in our patients. Moreover, the elevated level of IL-27 in the supernatant of ex vivo-cultured duodenal biopsies of untreated CD patients was found in the study of Di Sabatino et al. [43]. The authors discussed the possible involvement of IL-27 in the sustained Th1 response in untreated CD patients. Increased expression of IL-27 in active CD and its role in the inflammatory response and in the pathogenic mechanism of CD were shown in the study of Garrote et al. [22].

The level of TNF*α* was significantly higher in our CD patients compared to controls. The level of sTNFRII was also markedly elevated in the CD group and correlated with the degree of mucosal damage and the density of EV-positive cells in the *lamina propria.* There are data about the role of TNF in dysregulation of intestinal barrier permeability and tight junction disruption [63]. Significant elevation of TNF*α* was also found in the study of Manavalan et al. [13], where the monocyte-derived DCs from healthy donors were stimulated in vitro by gliadin. The increased number of cells stained for TNF*α* in the *lamina propria* and epithelium of untreated CD patients was reported in the study of Przemioslo et al. [19]. These authors emphasize the role of TNF*α* in the local immune response in the small bowel mucosa during active CD.

On the other hand, Wang et al. [63] showed that induction of TNFRII upregulation by IFN*γ* contributes to TNF-induced barrier dysfunction. The role of sTNFRII as an inhibitor of TNF and a naturally occurring damper of the inflammatory process in intestinal disease was also defined in the study of Noguchi et al. [64]. Our finding about the significant correlation of the sTNFRII level with the density of EV in the *lamina propria* in CD patients could reflect the possible involvement of sTNFRII in the immune response in the case of EV infection in their small bowel mucosa.

The role of IL-27 in the intestinal epithelial barrier maintenance and intestinal epithelial cell proliferation was demonstrated in the study of Diegelman et al. [65]. Additionally, IL-27 can induce indoleamine 2,3 dioxygenase (IDO) enzymatic activity, which leads to growth inhibition of intestinal bacteria by causing local tryptophan depletion. The activity of IDO stimulates development of anti-inflammatory regulatory T cells [65, 66]. In the present study, the IL-27 level was significantly correlated not only with IDO+ DCs but also with FOXP3+ Tregs. Thus, the increased level of IL-27 in our CD patients could be associated with EV infection, especially in those with coexisting T1D. Also, it might reflect the role of IL-27 in stimulating IDO expression and Treg differentiation. However, we could not rule out the role of other environmental factors, including recent reovirus as a candidate [3, 67] and other infectious agents [68].

In CD patients, as well as in CD patients with coexisting T1D, we noted a significantly elevated level of sIL-2R*α*, which showed correlation with the grade of small bowel mucosa damage according to Marsh classification. The elevated level of sIL-2R and good correlation with CD activity have been found in several studies [69–72]. A significant increase of intestinal permeability and sIL-2R concentration in patients with active CD was established in the study of Cummins et al. [69], and a correlation between IL-2R and the extent of mucosal damage according to the Marsh criterion was shown in the study of Kapoor et al. [72]. Additionally, sIL-2Ra was significantly correlated with the density of FOXP3+ Tregs and with IDO+ DCs in the *lamina propria* of the studied persons. As is known, the IL-2/IL-2 receptor system is critical for proper regulation of T cell proliferation [73]. Therefore, our result might support the evidence about the role of IL-2/IL-2R signals in peripheral maintenance of FOXP3+ Tregs [74, 75].

There is evidence that IP-10 plays a role in angiogenesis inhibition, cell chemotaxis, and regulation of cell growth, proliferation, and apoptosis [76, 77]. IP-10 is secreted by monocytes and endothelial cells in response to IFN*γ* [78]. Ontiveros et al. [79] reported that gluten peptide-stimulated production of IFN*γ* and IP-10 is specific for treated CD following gluten challenge. We found a significantly elevated level of IP-10 in CD patients, which correlated with the level of IFN*γ* and with the grade of small bowel mucosa damage according to the Marsh classification. This finding is consistent with the study of Bondar et al. [80], who demonstrated the increased production of CXCL10 (IP-10) in the epithelium and *lamina propria* of the intestinal mucosa of untreated CD patients while its expression was correlated with the IFN*γ* level in the tissue. The authors underline the role of the CXCL10/CXCR3 chemokine axis in immune cell recruitment in the inflamed small bowel mucosa of CD patients as a result of gluten action. Moreover, in our study, the level of IP-10 was significantly correlated with the density of EV-positive cells and the density of FOXP3+ Treg in the *lamina propria* of the small bowel mucosa in CD patients. There is evidence that CXCR3+ FOXP3 Tregs are able to migrate toward their chemokine ligand IP-10. This may facilitate their accumulation at the site of inflammation [81]. Ye et al. [48] showed that children with EV71 infection have enhanced IP-10 cytokine response in their cerebrospinal fluid, which indicates virus-induced inflammation and pathology.

The macrophage inflammatory protein *β* (MIP-1*β*), also known as CCL-4, belongs to the family of chemotactic cytokines produced by macrophages. We detected significantly higher MIP-1*β* levels in the groups of CD and CD with T1D. MIP-1*β* levels also correlated with the degree of small bowel mucosa damage as well as with the densities of CD103+ DCs and langerin+ DCs in the *lamina propria* of the small bowel mucosa. A significant increase of the MIP-1*β* (CCL-4) level in untreated CD was reported also in the study of Di Sabatino [43], which can be explained by recruitment of immune cells into the gut and driving the inflammatory response in CD [82]. Tucková et al. [83] reported that gliadin fragments were able to activate macrophages, which is consistent with increased levels of MIP-1*β* in our CD patients.

The significant correlation of the IL-7 level with the density of CD103+ DCs in the group of CD patients with coexisting T1D, shown in the present study, might reflect the role of IL-7 in the induction of expression of CD103+ DCs in the *lamina propria*. This supports our earlier suggestion that CD patients with associated T1D have stronger immune activation in the small intestinal mucosa compared to patients with CD alone [3]. The role of IL-7 in intestinal intraepithelial lymphocyte development and expression of CD103 (integrin *α*E*β*7) was shown in the study of Yang et al. [84].

In CD patients with T1D, the level of GM-CSF (granulocyte-macrophage colony-stimulating factor) was significantly correlated with the density of EV-positive cells in the *lamina propria*, which can support the evidence about the role of GM-CSF in intestinal homeostasis by maintaining the balance between tolerance of self-antigen and protection against pathogenic bacteria and also viruses including EV71 [58, 85]. GM-CSF may reinforce the intestinal mucosal barrier and increase neutrophil, monocyte, and macrophage activity [86, 87]. The study of Däbritz [88] underlined the role of GM-CSF in intestinal homeostasis by maintaining the integrity of the intestinal epithelium. According to Han et al. [89], neutralization of GM-CSF increased intestinal permeability and bacterial translocation in mice. The significant correlation of the GM-CSF level with the density of IDO+ DCs in our CD patients supports the role of GM-CSF in the regulation of the development of DCs [90].

There is evidence about the role of TGF*β*1 in maintaining immune homeostasis in the gut: in induction of Tregs, in differentiation of TH17 cells, and in generation of the IgA response [91, 92]. The significant correlation of the level of TGF*β*1 with the level of anti-tTG IgA in our CD patients supports this statement. Significant enhancement of expression of TGF*β*1 and tissue transglutaminase in the small intestine of children with CD was evaluated in the study of Hansson et al. [93]. The study of Benahmed et al. [94] showed the inhibitory effect of IL-15 on TGF-*β* signaling, which leads to promotion of intestinal inflammation in CD. In our study, the levels of TGF*β*1 and TGF*β*2 were significantly lower compared with those of controls. Moreover, a significant negative correlation between TGF*β*2 and IL-15 levels was found for CD patients, which may support the finding of Benahmed et al. [94].

The levels of IL-5 and IL-13 cytokines involved in the Th2 cell response were significantly increased in CD patients. In contrast, the level of IL-4 was significantly lower in our CD patients compared with controls.

IL-5 level was also correlated with the degree of small bowel mucosa damage. Similar to our results, Björck et al. [23] found significant elevation of these cytokines in the serum of CD children. IL-5 plays a major role in eosinophilic recruitment and has been found in the jejunal mucosa of patients with CD and *dermatitis herpetiformis* [95].

Previous studies have shown the role of IL-13 in gut defense and inflammation [96] and a toxic effect on colonic epithelial cells and on the epithelial barrier in ulcerative colitis [97]. Thus, our results and those of other researchers clearly suggest that, in addition to the prevailing Th1 and Th17 cell activation mechanisms, also Th2 cells in association with other immunoregulatory mechanisms (Treg etc.) are important players in the pathogenesis of CD.

We took account of the possible influence of the seasons on the level of cytokines. However, the seasonal difference in the cytokine level was noticed for CD patients only for IL-22 which was significantly higher in spring compared to summer. A major impact of seasonality on cytokine response and inflammation was reported in the study of ter Horst et al. [98]. These authors detected a significant peak in the production of TNF*α*, IL-1*β*, and IL-6 in summer.

The possible influence of gender on the cytokine level was also considered in our study. We noted significantly higher levels of IL-1*β*, IL-2, IL-8, IL-12 (P70), and leptin in females compared to males. At the same time, a significantly higher level of TGF*β*1 occurred exclusively among males. Significantly higher serum levels of TGF*β*1 in men compared to women in healthy controls but similar levels in both male and female patients with autoimmune diseases were also found in the study of Manolova et al. [99].

Higher concentrations of leptin in women compared to men have also been reported in several other studies [97, 100]. The higher production rate of leptin per unit mass of the adipose tissue and a larger adipose tissue mass in women versus men is considered to be the reason for gender differences in circulating leptin levels [100]. Although in our study, the patients and the controls were children, the level of leptin was higher in girls and it increased with age, which supports the finding about gender differences in adiponectin levels during the progression of puberty [101].

In our study, a significantly higher serum level of IL-8 was found among females compared to males. Although there is no good general explanation for this finding in the literature, Bendrik and Dabrosin [102] have demonstrated that estrogen can have a stimulating effect on IL-8 production by immune cells, which might also explain our results.

We found the level of leptin alone to be positively correlated with age, whereas the levels of IL-8, IP-10, MIP-1*β*, sIL-2R*α*, TGF*β*3, MCP-1, TNF*α*, and GM-CSF decreased with increasing age. This could be related to the fact that our control persons were significantly older than CD patients, and hence, the levels of IL-8, IP-10, MIP-1*β*, sIL-2R*α*, and TNF*α* were lower in controls than in CD patients. Ageing-associated decrease in chemotaxis, GM-CSF signaling, and TLR-induced cytokine production, as well as cytokine production by macrophages, has been established by several authors [103, 104]. However, we have kept in mind that our study group consisted of children (median age 10.1 years) and not elderly persons investigated in the abovementioned studies.

Our study had several limitations, such as the relatively small number of the persons studied. In addition, our control group did not include healthy persons but consisted of patients with FGD, duodenal ulcer, and erosive gastritis, as we could not investigate healthy persons using gastroduodenoscopy, for ethical considerations, nor could we study cytokines in the intestinal mucosa, due to the shortage of intestinal mucosa samples. The number of biopsy samples was limited because of ethical constraints. On the other hand, the strength of the study was the circumstance that we investigated a wide panel of cytokines with a simultaneous evaluation of immune cell subpopulations of the small bowel mucosa in the same patients. Importantly, we evaluated associations between cytokines and EV in patients with CD, as well as in patients with CD and T1D.

## 5. Conclusions

Taken together, the most important finding of the study is the significant increase of several proinflammatory cytokines and chemokines like IL-15, IL-8, IL-17F, IP-10, and MIP-1*β* in CD patients and its highly significant correlation with the grade of small bowel mucosa atrophy, which plays a major role as a sign of small bowel mucosa inflammation and damage. CD patients with coexisting T1D showed particularly high levels of IL-15, IL-17F, MIP-1*β*, and sIL-2R*α* compared to the control group.

Additionally, several cytokines like IL-2, IL-6, IL-12 (P70), IL-15, IP-10, and IFN*γ* correlated with the density of FOXP3+ Tregs in the *lamina propria* of the small bowel mucosa, which supports the evidence about the signaling of these cytokines in peripheral maintenance of FOXP3+ Tregs. An important observation of the study was the correlation of IL-17F, IP-10, sTNFRII, MCP-1, and GM-CSF with the density of EV-positive cells in the *lamina propria* of the small bowel mucosa, which suggests the predominance of the Th1 and Th17 immune responses to environmental factors like viral infection in CD and T1D patients.

Correlation of MIP-1*β* (CCL-4) with CD103+ DCs and langerin+ DCs may indicate recruitment of immune cells into the *lamina propria* of the small bowel mucosa and driving of the inflammatory response in CD patients.

Another important finding of our study was also the significant elevation of Th2 cytokines like IL-5 and IL-13 in CD patients and its correlation with the degree of small bowel mucosa damage, suggesting the possible role of Th2 cell-related mechanisms in the pathogenesis of CD. To sum up, our study demonstrates that CD, but particularly CD with accompanying T1D, is characterized by substantial changes in the blood cytokine balance, which may be related to inflammatory processes in the small intestinal mucosa.

## Figures and Tables

**Table 1:** Number, median age, gender, and median values of IgA to tTG of the study persons.

Study groups	CD		CD + T1D		All CDs		T1D		Control group	
Gender	Male	Female	Male	Female	Male	Female	Male	Female	Male	Female
Number of persons	*n* = 8	*n* = 16^∗^	*n* = 6	*n* = 3^∗∗^	*n* = 14	*n* = 19^∗∗∗^	*n* = 2	*n* = 0	*n* = 12	*n* = 25^∗∗∗∗^
Median age (years) (IQR)	7.0 (3.5-19.7)	6.5^#^ (5.0-13.3)	7.0 (3.4-11.6)	7.0 (6.0-19.4)	7.0 (3.4-10.1)	7.0^##^ (5.0-11.0)	8.5 (4.0-13.1)		13.5 (4.9-15.7)	14.9^###^ (3.0-16.7)
IgA-tTG median value (EIU) (IQR)	140.0 (108.8-549.6)	115.5 (78.5-418.8)	126.5 (78.5-1693)	52.3 (35.0-28.0)	134.5 (102.5-725)	103.00 (52.3-400)	8.4 (7.0-9.8)		0.30 (0.1-1.2)	0.65 (0.4-1.1)

CD: celiac disease; T1D: type 1 diabetes; IQR: interquartile range (25%-75%); EIU: enzyme immunoassay units (values of IgA-tTG higher than 10 EliA U/ml are considered positive). No significant difference between the number of males and the number of females studied in the CD group: ^∗^ *χ* ^2^ = 1.8, *P* = 0.17; in the CD + T1D group: ^∗∗^ *χ* ^2^ = 0.67, *P* = 0.41; in the whole CD group: ^∗∗∗^ *χ* ^2^ = 0.50, *P* = 0.47; and in the control group: ^∗∗∗∗^ *χ* ^2^ = 3.08, *P* = 0.07. Significantly higher median age of females in the control group compared to median age of females in the CD group (^###^ > ^#^ *P* = 0.009) and in all CDs (^###^ > ^##^ *P* = 0.004) (Mann-Whitney *U* test). No significant difference between the median age of males and that of females in the CD group (*P* = 0.55), in the CD + T1D group (*P* = 0.79), in the whole CD group (*P* = 0.51), and in the control group (*P* = 0.48) (Mann-Whitney *U* test). No significant difference between the median values of IgA to tTG between males and females in the CD group (*P* = 0.37), in the CD + T1D group (*P* = 0.26), in the whole CD group (*P* = 0.17), and in the control group (*P* = 0.16) (Mann-Whitney *U* test).

**Table 2:** Seasonal distribution of samplings between the study groups.

Study group	Seasons				
	Winter	Spring	Summer	Autumn	Total
All CDs	12	5^∗^	6	10	33
Normal mucosa	9	12	5	13	39
Total	21	17	11^∗∗^	23	72

Between all groups: *χ* ^2^ = 3.66, *P* = 0.30; ^∗^between all CDs and normal mucosa: *χ* ^2^ = 4.23, *P* = 0.039 with Yates's correction; ^∗∗^between winter and summer: *χ* ^2^ = 4.0, *P* = 0.04; between summer and autumn: *χ* ^2^ = 5.55, *P* = 0.01.

**Table 3:** Cytokine levels (median values (IQR)) in different study groups.

Cytokine	Controls	CD	CD + T1D	All CD cases	T1D	*P* values	Groups compared
*n*	37	24	9	33	2		
IL-1*β* (pg/ml)	2.76 (1.6-3.2)	2.59 (2.25-3.25)	2.39 (1.5-3.21)	2.45 (1.8-3.2)	2.92 (2.3-3.5)	*P* > 0.05	Between all groups
IL-2 (pg/ml)	1.97 (0.69-3.27)	0.95 (0.04-2.42)	1.57 (0.51-4.15)	1.53 (0.22-2.81)	2.62 (1.88-3.35)	*P* > 0.05	Between all groups
IL-4 (pg/ml)	54.7 (27.0-90.1)	18.1 (3.6-64.6)	18.3 (12.4-52.7)	18.3 (6.8-55.3)	20.5 (14.4-26.54)	*P* = 0.01	Controls vs CD
IL-5 (pg/ml)	4.26 (3.2-5.7)	6.0 (4.5-9.7)	5.82 (4.2-7.8)	5.82 (4.5-8.7)	11.4 (5.2-17.6)	*P* = 0.007	CD vs controls
IL-6 (pg/ml)	1.81 (1.44-2.83)	2.22 (1.79-3.36)	2.78 (1.36-4.98)	2.45 (1.75-3.35)	1.9 (1.60-2.23)	*P* > 0.05	Between all groups
IL-7 (pg/ml)	10.89 (8.8-12.3)	9.7 (8.4-10.8)	11.27 (8.8-12.0)	9.81 (8.4-11.3)	21.34 (11.7-31.3)	*P* > 0.05	Between all groups
IL-8 (pg/ml)	13.5 (8.5-19.9)	30.1 (15.3-58.8)	21.2 (10.9-52.2)	24.8 (14.5-43.7)	17.91 (17.8-17.5)	*P* < 0.0001	CD vs controls
IL-10 (pg/ml)	11.3 (7.08-18.1)	14.2 (9.0-19.3)	19.2 (11.0-22.8)	14.3 (9.65-20.6)	21.98 (9.5-34.3)	*P* > 0.05	Between all groups
IL-12 (P70) (pg/ml)	7.2 (5.98-9.38)	7.6 (4.7-8.9)	5.83 (3.1-8.5)	7.1 (4.6-8.9)	8.2 (7.6-8.8)	*P* > 0.05	Between all groups
IL-13 (pg/ml)	12.5 (8.2-20.6)	19.3 (11.0-33.8)	21.32 (11.5-41.1)	20.32 (11.2-36.3)	46.2 (18.7-73.7)	*P* = 0.02	All CDs vs controls
IL-15 (pg/ml)	0.0 (0.0-0.0.)	6.06 (0.0-11.4)	6.03 (0.0-13.7)	6.05 (0.0-11.7)	26.6 (12.3-4.08)	*P* < 0.0001 *P* = 0.006	CD vs controls CD + T1D vs controls
IL-17A (pg/ml)	14.54 (9.35-22.3)	12.1 (7.9-14.7)	12.5 (7.2-22.6)	12.25 (7.5-17.5)	24.21 (9.7-38.7)	*P* > 0.05	Between all groups
IL-17F (ng/ml)	0.0 (0.0-0.0)	0.004 (0.0-0.05)	0.02 (0.0-0.10	0.008 (0.0-0.06)	0.20 (0.01-0.38)	*P* = 0.02 *P* = 0.03 *P* = 0.008	CD vs controls CD + T1D vs controls All CDs vs controls
IL-21 (pg/ml)	2.74 (1.33-4.3)	2.08 (1.23-4.4)	2.17 (0.65-3.8)	2.17 (1.1-4.7)	12.92 (2.4-23.4)	*P* > 0.05	Between all groups
IL-22 (ng/ml)	0.0 (0.0-0.05)	0.38 (0.0-1.08)	0.0 (0.0-1.5)	0.30 (0.0-1.05)	2.12 (0.29-3.95)	*P* = 0.009	CD vs controls
IL-23 (pg/ml)	429.5 (219.5-803.7)	347.5 (241.3-837.9)	228.6 (172.2-754.0)	340.0 (210.2-820.3)	826.9 (366.5-1287)	*P* > 0.05	Between all groups
IL-27 (ng/ml)	0.75 (0.56-1.23)	1.75 (1.27-3.39)	1.0 (0.66-4.09)	1.72 (0.99-3.24)	7.84 (1.0-14.6)	*P* < 0.0001	CD vs controls
IP-10 (pg/ml)	189.3 (122.5-304.1)	254.9 (196.3-378.4)	243.9 (174.1-305.3)	247.1 (185.8-340.8)	417.2 (195.2-639.2)	*P* = 0.012	CD vs controls
IFN*γ* (pg/ml)	20.08 (13.2-25.2)	20.63 (11.25-27.53)	17.15 (15.34-33.83)	18.69 (12.60-28.56)	48.33 (14.95-81.70)	*P* > 0.05	Between all groups
MIP-1*β* (pg/ml)	35.46 (21.43-58.14)	51.44 (38.33-73.03)	52.85 (44.69-70.2)	52.85 (41.7-71.26)	81.57 (48.66-114.5)	*P* = 0.02 *P* = 0.02 *P* = 0.005	CD vs controls CD + T1D vs controls All CDs vs controls
MCP1 (pg/ml)	613.6 (489.0-742.0)	563.9 (513.9-813.1)	698.7 (463.9-937.3)	607.1 (516.3-845.4)	925.8 (664.5-1187)	*P* > 0.05	Between all groups
TNF*α* (pg/ml)	10.19 (7.4-13.4)	13.09 (9.45-18.8)	10.61 (9.7-16.9)	12.62 (9.71-18.48)	16.91 (12.07-21.75)	*P* = 0.03	CD vs controls
PAI-1 (pg/ml)	521334 (347801-667527)	214127 (124009-564711)	496513 (238994-689119)	379704 (128294-648666)	869655 (691851 − 1047*E* + 006)	*P* = 0.01	Controls vs CD
TGF*β*1 (pg/ml)	58013 (46296-64451)	46973 (34771-55086)	56933 (42377-69830)	48378 (37144-58561)	76385 (58782-93988)	*P* = 0.004	Controls vs CD
TGF*β*2 (pg/ml)	1840 (1521-2227)	1481 (1148-1719)	1697 (1347-2082)	1574 (1264-1949)	2165 (1791-2538)	*P* = 0.006	Controls vs CD
TGF*β*3 (pg/ml)	12.33 (6.16-122.2)	29.09 (12.33-92.02)	122.2 (12.33-130.2)	29.09 (12.33-122.2)	61.11 (0.0-122.2)	*P* > 0.05	Between all groups
sIL-1R1 (pg/ml)	27.72 (25.3-34.4)	0 (0.0-27.7)	25.3 (18.67-30.14)	21.68 (0.0-27.72)	36.84 (25.30-48.38)	*P* = 0.0008	Controls vs CD
sIL-2R*α* (pg/ml)	614.3 (456.3-754.1)	971.7 (759.5-1464)	923.7 (757.2-1245)	952.7 (784.2-1394)	1448 (1213-1684)	*P* = 0.0003 *P* = 0.005	CD vs controls CD + T1D vs controls
sTNFRII (pg/ml)	5928 (5142-7106)	7445 (6257-9256)	5748 (4609-8367)	7336 (5746-8582)	9744 (8755-10733)	*P* = 0.005 *P* = 0.03	CD vs controls T1D vs controls
GM-CSF (pg/ml)	123.1 (69.1-209.2)	155.4 (51.85-231.8)	106.4 (86.3-249.5)	154.4 (62.51-232.6)	131.1 (70.65-191.6)	*P* > 0.05	Between all groups
Adiponectin (pg/ml)	1.6*E* + 008 (1.6*E* + 008 − 1.6*E* + 008)	1.6*E* + 008 (1.18*E* + 008 − 1.6*E* + 008)	1.6*E* + 008 (1.6*E* + 008 − 1.6*E* + 008)	1.6*E* + 008 (1.57*E* + 008 − 1.6*E* + 008)	1.6*E* + 008 (1.6*E* + 008 − 1.6*E* + 008)	*P* > 0.05	Between all groups
Leptin (pg/ml)	2752 (1246-73621)	1260 (863.6-27469	765.6 (521.4-3790)	1152 (768.0-2913)	1316 (1178-1454)	*P* = 0.03	Controls vs CD
Resistin (pg/ml)	259209 (199695-348865)	191157 (91529-347211)	207472 (151595-400718)	206244 (136354-370681)	293281 (181429-405134)	*P* > 0.05	Between all groups

Median values between the groups were compared using the Mann-Whitney *U* test.

## Data Availability

The data used to support the findings of this study are available from the corresponding author Tamara Vorobjova upon request.

## Supplementary Materials

Supplementary Materials Supplementary Table 1: minimum and maximum detectable concentrations of analyzed cytokines detected by Milliplex® MAP Magnetic Bead assays. Supplementary Table 2: CD- and T1D-associated HLA-DR/DQ haplotypes in studied patients (deduced by genotyping data according to Ilonen et al. [35]). Supplementary Table 3: results of the comparison of cytokine levels between CD patients and control persons evaluated in patients' sera. Supplementary Table 4: results of multiple regression analysis (independent variables: CD and T1D diagnoses, age, gender, and season). Supplementary Table 5: ^∗^significant correlations between cytokine levels and markers for Tregs, DCs, and EV in the study groups.
