# Supplementary material for: Celiac Disease in Children, Particularly with Accompanying Type 1 Diabetes, Is Characterized by Substantial Changes in the Blood Cytokine Balance, Which May Reflect Inflammatory Processes in the Small Intestinal Mucosa
Source: J Immunol Res. 2019 May 12;2019:6179243. doi: 10.1155/2019/6179243 (PMC6535873; doi:10.1155/2019/6179243)
Supplement: Supplementary Materials — Supplementary Table 1: minimum and maximum detectable concentrations of analyzed cytokines detected by Milliplex® MAP Magnetic Bead assays. Supplementary Table 2: CD- and T1D-associated HLA-DR/DQ haplotypes in studied patients (deduced by genotyping data according to Ilonen et al. [35]). Supplementary Table 3: results of the comparison of cytokine levels between CD patients and control persons evaluated in patients' sera. Supplementary Table 4: results of multiple regression analysis (independent variables: CD and T1D diagnoses, age, gender, and season). Supplementary Table 5: ∗significant correlations between cytokine levels and markers for Tregs, DCs, and EV in the study groups. [file 6179243.f1.docx]

Supplementary Table 1: Minimum and maximum detectable concentrations of analyzed cytokines detected by Milliplex® MAP Magnetic Bead assays.

| **Cytokine** | **Kit name** | **Minimal detectable concentration*** | **Maximal detectable concentration*** | **Sample dilution** |
| --- | --- | --- | --- | --- |
| Adiponectin | Human Adipokine 1 | 26 pg/ml | 400000 pg/ml | 1/400 |
| Resistin | Human Adipokine 1 | 6.4 pg/ml | 100000 pg/ml | 1/400 |
| PAI-1 | Human Adipokine 1 | 9.6 pg/ml | 150000 pg/ml | 1/400 |
| Leptin | Human Adipokine 2 | 38 pg/ml | 600000 pg/ml | undiluted |
| IP-10 | Human Cytokine/Chemokine | 3.2 pg/ml | 10000 pg/ml | undiluted |
| MCP-1 | Human Cytokine/Chemokine | 3.2 pg/ml | 10000 pg/ml | undiluted |
| MIP-1β | Human Cytokine/Chemokine | 3.2 pg/ml | 10000 pg/ml | undiluted |
| GM-CSF | Human High Sensitivity T Cell | 8.25 pg/ml | 5000 pg/ml | undiluted |
| IFN-γ | Human High Sensitivity T Cell | 0.61 pg/ml | 2500 pg/ml | undiluted |
| IL-10 | Human High Sensitivity T Cell | 1.46 pg/ml | 6000 pg/ml | undiluted |
| IL-12(p70) | Human High Sensitivity T Cell | 0.49 pg/ml | 2000 pg/ml | undiluted |
| IL-13 | Human High Sensitivity T Cell | 0.24 pg/ml | 1000 pg/ml | undiluted |
| IL-17A | Human High Sensitivity T Cell | 0.73 pg/ml | 3000 pg/ml | undiluted |
| IL-1b | Human High Sensitivity T Cell | 0.49 pg/ml | 2000 pg/ml | undiluted |
| IL-2 | Human High Sensitivity T Cell | 0.49 pg/ml | 2000 pg/ml | undiluted |
| IL-21 | Human High Sensitivity T Cell | 0.24 pg/ml | 1000 pg/ml | undiluted |
| IL-4 | Human High Sensitivity T Cell | 1.83 pg/ml | 7500 pg/ml | undiluted |
| IL-23 | Human High Sensitivity T Cell | 7.93 pg/ml | 32500 pg/ml | undiluted |
| IL-5 | Human High Sensitivity T Cell | 0.49 pg/ml | 2000 pg/ml | undiluted |
| IL-6 | Human High Sensitivity T Cell | 0.18 pg/ml | 750 pg/ml | undiluted |
| IL-7 | Human High Sensitivity T Cell | 0.37 pg/ml | 1500 pg/ml | undiluted |
| IL-8 | Human High Sensitivity T Cell | 0.31 pg/ml | 1250 pg/ml | undiluted |
| TNFα | Human High Sensitivity T Cell | 0.43 pg/ml | 1750 pg/ml | undiluted |
| sIL-1RI | Human Soluble Cytokine Receptor | 24.4 pg/ml | 100000 pg/ml | 1/5 |
| sIL-2Rα | Human Soluble Cytokine Receptor | 24.4 pg/ml | 100000 pg/ml | 1/5 |
| sTNFRII | Human Soluble Cytokine Receptor | 12.2 pg/ml | 50000 pg/ml | 1/5 |
| IL-17F | Human Th17 | 0.02 ng/ml | 100 ng/ml | undiluted |
| IL-15 | Human Th17 | 5 pg/ml | 20000 pg/ml | undiluted |
| IL-22 | Human Th17 | 0.04 ng/ml | 150 ng/ml | undiluted |
| IL-27 | Human Th17 | 0.06 ng/ml | 250 ng/ml | undiluted |
| TGFβ1 | TGF-β 1,2,3 | 9.8 pg/ml | 10000 pg/ml | 1/30 |
| TGFβ2 | TGF-β 1,2,3 | 9.8 pg/ml | 10000 pg/ml | 1/30 |
| TGFβ3 | TGF-β 1,2,3 | 9.8 pg/ml | 10000 pg/ml | 1/30 |

* Calculated according to the Millipore Magnetic Bead Panel kit’s protocols.

Supplementary Table 2: CD and T1D associated HLA-DR/DQ haplotypes in studied patients (deduced by genotyping data according to Ilonen et al. [35])

| Patients studied | Haplotype 1 | Haplotype 2 |
| --- | --- | --- |
| CD | DR3-DQ2 | - |
| CD | DR3-DQ2 | - |
| CD | DR3-DQ2 | - |
| CD | DR3-DQ2 | - |
| CD | DR3-DQ2 | - |
| CD | DR3-DQ2 | - |
| CD | DR3-DQ2 | DR3-DQ2 |
| CD | DR3-DQ2 | - |
| CD | DR3-DQ2 | - |
| CD | DR3-DQ2 | - |
| CD+T1D | DR3-DQ2 | DR4-DQ8 |
| CD+T1D | DR4-DQ8 | - |
| CD+T1D | DR3-DQ2 | - |
| CD+T1D | DR3-DQ2 | DR4-DQ8 |
| CD+T1D | DR4-DQ8 | - |
| CD+T1D | DR3-DQ2 | DR3-DQ2 |
| T1D | DR3-DQ2 | DR4-DQ8 |

Supplementary Table3: Results of the comparison of cytokine levels between CD patients and control persons evaluated in patients’sera.

| Cytokines whose level was significantly higher **in** **CD** patients compared to controls | | Cytokines whose level was significantly higher  **in CD +T1D** patients compared to controls | | Cytokines whose level was significantly higher in **control persons** compared to  CD patients | | Cytokines whose level did not differ between study groups | |
| --- | --- | --- | --- | --- | --- | --- | --- |
| Cytokine | *p* * | Cytokine | *p* * | Cytokine | *p* * | Cytokine | *p* ** |
| IL-5 | 0.007 | IL-15 | 0.006 | IL-4 | 0.01 | IL-1β | 0.72 |
| IL-8 | <0.0001 | IL-17F | 0.03 | PAI-1 | 0.01 | IL-2 | 0.50 |
| IL-13 | 0.02 (all CD)*** | MIP-1β | 0.02 | TGFβ1 | 0.004 | IL-6 | 0.29 |
| IL-15 | <0.0001 | sIL-2Rα | 0.005 | TGFβ2 | 0.006 | IL-7 | 0.26 |
| IL-17F | 0.02/ 0.008 (all CD) |  |  | sIL-1R1 | 0.0008 | IL-10 | 0.28 |
| IL-22 | 0.009 |  |  | Leptin | 0.03 | IL-12(P70) | 0.37 |
| IL-27 | <0.0001 |  |  |  |  | IL-17A | 0.34 |
| IP-10 | 0.012 |  |  |  |  | IL-21 | 0.60 |
| MIP-1β | 0.02/0.005 (all CD) |  |  |  |  | IL-23 | 0.67 |
| sIL-2Rα | 0.0003 |  |  |  |  | IFN-γ | 0.96 |
| sTNFRII | 0.005 |  |  |  |  | MCP-1 | 0.73 |
| TNFα | 0.03 |  |  |  |  | GM-CSF | 0.75 |
|  |  |  |  |  |  | TGFβ3 | 0.24 |
|  |  |  |  |  |  | Adiponectin | 0.72 |
|  |  |  |  |  |  | Resistin | 0.23 |

*p** values were determined using the Mann-Whitney test.

*p*** values were determined using the Kruskal –Wallis test (comparing three independent study groups: CD, CD+T1D and control persons with normal small bowel mucosa).

***** (**all CD) p value determined comparing all CD group with controls.

Supplementary Table 4: Results of multiple regressioon analysis (Independent variables: CD and T1D diagnose, age, gender, season)

| Dependent cytokine | CD and T1D Diagnose | | Age | | Gender | | Season | |
| --- | --- | --- | --- | --- | --- | --- | --- | --- |
|  | R  partial | p | R  partial | p | R  partial | p | R partial | p |
| IL-1β | 0.03 | 0.77 | -0.02 | 0.81 | -0.29 | 0.01 | 0.03 | 0.77 |
| IL-2 | 0.02 | 0.81 | 0.09 | 0.45 | -0.24 | 0.04 | -0.15 | 0.20 |
| IL-4 | -0.24 | 0.04 | 0.08 | 0.50 | 0.04 | 0.71 | 0.05 | 0.64 |
| IL-5 | 0.33 | 0.005 | -0.07 | 0.55 | -0.18 | 0.12 | 0.02 | 0.85 |
| IL-6 | 0.03 | 0.80 | -0.17 | 0.14 | -0.02 | 0.82 | 0.19 | 0.11 |
| IL-7 | 0.10 | 0.38 | 0.17 | 0.16 | -0.04 | 0.69 | 0.03 | 0.79 |
| IL-8 | 0.27 | 0.02 | -0.18 | 0.13 | -0.30 | 0.01 | -0.06 | 0.60 |
| IL-10 | 0.06 | 0.60 | -0.08 | 0.51 | -0.16 | 0.16 | -0.23 | 0.05 |
| IL-12 (p70) | -0.05 | 0.66 | 0.18 | 0.12 | -0.28 | 0.01 | 0.01 | 0.89 |
| IL-13 | 0.35 | 0.003 | -0.02 | 0.85 | -0.05 | 0.63 | -0.11 | 0.35 |
| IL-15 | 0.16 | 0.17 | 0.07 | 0.53 | 0.13 | 0.25 | -0.16 | 0.16 |
| IL-17A | 0.05 | 0.65 | 0.05 | 0.64 | -0.15 | 0.20 | -0.009 | 0.93 |
| IL-17F | 0.12 | 0.30 | 0.02 | 0.85 | 0.15 | 0.20 | -0.21 | 0.07 |
| IL-21 | -0.002 | 0.98 | 0.11 | 0.36 | 0.08 | 0.47 | 0.18 | 0.12 |
| IL-22 | 0.21 | 0.07 | 0.03 | 0.80 | 0.13 | 0.25 | -0.23 | 0.04 |
| IL-23 | 0.08 | 0.47 | 0.08 | 0.50 | -0.18 | 0.12 | -0.01 | 0.87 |
| IL-27 | 0.12 | 0.32 | 0.03 | 0.79 | 0.14 | 0.23 | -0.19 | 0.09 |
| IP-10 | 0.22 | 0.06 | 0.40 | 0.0005 | 0.07 | 0.55 | -0.08 | 0.49 |
| IFNγ | 0.07 | 0.51 | -0.04 | 0.72 | -0.01 | 0.88 | -0.17 | 0.15 |
| MIP-1β | 0.26 | 0.03 | -0.32 | 0.006 | 0.02 | 0.80 | -0.14 | 0.23 |
| MCP-1 | 0.0.08 | 0.50 | -0.23 | 0.05 | 0.12 | 0.31 | -0.06 | 0.58 |
| TNFα | 0.04 | 0.68 | -0.32 | 0.005 | -0.0003 | 0.99 | -0.0007 | 0.99 |
| PAI-1 | -0.11 | 0.36 | 0.005 | 0.96 | 0.22 | 0.06 | -0.12 | 0.31 |
| TGFβ1 | -0.08 | 0.50 | -0.07 | 0.53 | 0.24 | 0.04 | -0.05 | 0.63 |
| TGFβ2 | -0.15 | 0.20 | -0.14 | 0.22 | 0.16 | 0.16 | -0.09 | 0.44 |
| TGFβ3 | 0.07 | 0.54 | -0.28 | 0.01 | -0.19 | 0.11 | 0.004 | 0.96 |
| sIL-1R1 | -0.22 | 0.06 | 0.09 | 0.45 | 0.27 | 0.02 | -0.15 | 0.21 |
| sIL-2Rα | 0.03 | 0.75 | -0.27 | 0.02 | 0.22 | 0.07 | -0.06 | 0.63 |
| sTNFRII | 0.18 | 0.14 | 0.12 | 0.32 | 0.17 | 0.17 | -0.08 | 0.49 |
| GM-CSF | 0.003 | 0.97 | -0.18 | 0.12 | -0.17 | 0.14 | 0.15 | 0.20 |
| Adiponectin | -0.03 | 0.75 | -0.15 | 0.20 | -0.02 | 0.85 | -0.16 | 0.16 |
| Leptin | 0.11 | 0.36 | 0.68 | <0.0001 | -0.32 | 0.006 | 0.10 | 0.37 |
| Resistin | 0.03 | 0.77 | 0.07 | 0.56 | -0.12 | 0.30 | -0.10 | 0.36 |

**r _partial_** – the partial correlation coefficient is the coefficient of correlation of the variable with the dependent variable, adjusted for the effect of the other variables in the model. If p is less than the conventional 0.05, the regression coefficient can be considered to be significantly different from 0, and the corresponding variable contributes significantly to the prediction of the dependent variable. (MedCalc statistic).

Supplementary Table 5: Significant **c**orrelations* between cytokine levels and markers for Tregs, DCs, EV in the study groups

| Cytokine | Study groups | Density of FOXP3+ Tregs | Density of IDO+ DCs | CD11c+ DCs density | CD103+ DCs density | Langerin (CD207)+ DCs density | EV+ cell density | Anti- EV IgA level | Anti- EV  IgG level |
| --- | --- | --- | --- | --- | --- | --- | --- | --- | --- |
| IL-1β | Controls  CD  CD+T1D  All CD case |  | . |  |  |  |  | . | . |
| IL-2 | Controls  CD  CD+T1D  All CD cases | R=0.54 p=0.02  R=0.48 p=0.01 |  |  |  | R=0.38 p=0.02 |  |  |  |
| IL-4 | Controls  CD  CD+T1D  All CD cases | R=-0.50 p=0.004 | R=-0.40 p=0.02  R=-0.58 p=0.02. |  |  |  | R=-0.36 p=0.04 |  |  |
| IL-5 | Controls  CD  CD+T1D  All CD cases |  |  |  |  |  |  |  |  |
| IL-6 | Controls  CD  CD+T1D  All CD cases | R=0.65 p=0.006 |  |  |  |  |  |  |  |
| IL-7 | Controls  CD  CD+T1D  All CD cases |  |  |  | R=0.85 p=0.01 | R=0.38 p=0.02 |  |  |  |
| IL-8 | Controls  CD  CD+T1D  All CD cases |  |  |  |  |  |  |  |  |
| IL-10 | Controls  CD  CD+T1D  All CD cases |  | R=0.56 p=0.02 | R=0.59 p=0.0006 |  |  |  |  |  |
|  |  |  |  |  |  |  |  |  |  |
| Cytokine | Study groups | Density of FOXP3+ Tregs | Density of IDO+ DCs | CD11c+ DCs density | CD103+ DCs density | Langerin (CD207+ DCs density | EV+ cell density | Anti EV IgA level | Anti EV IgG level |
| IL-12 (P70) | Controls  CD  CD+T1D  All CD cases | R=0.66 p=0.005 | R=0.57 p=0.02 | R=0.59 p=0.01  R=0.44 p=0.03 |  | R=0.40 p=0.01 |  |  |  |
| IL-13 | Controls  CD  CD+T1D  All CD cases |  |  |  |  |  |  |  |  |
| IL-15 | Controls  CD  CD+T1D  All CD cases | R=0.76 p=0.04 |  |  |  |  |  |  |  |
| IL-17A | Controls  CD  CD+T1D  All CD cases  Whole study group |  |  | R=0.56 p=0.02 |  |  |  |  |  |
| IL-17F | Controls  CD  CD+T1D  All CD cases |  |  | R=0.40 p=0.02 |  |  | R=0.36 p=0.04 |  | R=0.93 p=0.001  R=0.62 p=0.005 |
| IL-21 | Controls  CD  CD+T1D  All CD cases |  |  | R=0.59 p=0.01 |  |  |  |  |  |
| IL-22 | Controls  CD  CD+T1D  All CD cases |  |  | R=0.48 p=0.006 |  |  |  |  |  |
| IL-23 | Controls  CD  CD+T1D  All CD cases |  |  | R=0.42 p=0.04 |  | R=0.45 p=0.006 |  |  | R=0.73 p=0.04  R=0.50 p=0.03 |
| IL-27 | Controls  CD  CD+T1D  All CD cases |  |  | R=0.36 p=0.04 |  |  |  |  | R=0.95 p=0.001 |
| IP-10 | Controls  CD  CD+T1D  All CD cases | R=0.68 p=0.003 | R=0.43 p=0.0009 | R=0.51 p=0.003 |  |  | R=0.52 p=0.04 |  |  |
| Cytokine | Study groups | Density of FOXP3+ Tregs | Density of IDO+ DCs | CD11c+ DCs density | CD103+ DCs density | Langerin (CD207+ DCs density | EV+ cell density | Anti EV IgA level | Anti EV IgG level |
| INF-γ | Controls  CD  CD+T1D  All CD cases | R=0.56 p=0.02 | R=0.53 p=0.04 |  |  |  |  |  |  |
| MIP-1β | Controls  CD  CD+T1D  All CD cases |  |  |  | R=0.42 p=0.03 | R=0.52 p=0.02  R=0.56 p=0.003 |  |  |  |
| MCP1 | Controls  CD  CD+T1D  All CD cases |  |  |  |  |  | R=-0.45 p=0.002 |  |  |
| TNFα | Controls  CD  CD+T1D  All CD cases |  |  |  |  |  |  |  |  |
| PAI-1 | Controls  CD  CD+T1D  All CD cases |  | R=-0.50 p=0.05 |  |  |  |  |  |  |
| TGFβ1 | Controls  CD  CD+T1D  All CD cases | R=-0.85 p=0.02  R=-0.47 p=0.02 | R=-0.73 p=0.04  R=-0.54 p=0.01 |  |  |  | R=-0.63 p=0.01  R=-0.50 p=0.013 |  |  |
| TGFβ2 | Controls  CD  CD+T1D  All CD cases |  |  |  |  | R=0.36 p=0.03 |  |  |  |
| TGFβ3 | Controls  CD  CD+T1D  All CD cases |  |  |  |  |  |  |  |  |
| sIL-1R1 | Controls  CD  CD+T1D  All CD cases |  | R=-0.68 p=0.004 |  |  |  | R=-0.50 p=0.01 |  |  |
| sIL-2Rα | Controls  CD  CD+T1D  All CD cases | R=0.46 p=0.009 | R=0.39 p=0.03 | R=0.41 p=0.02 |  |  |  |  |  |
| Cytokine | Study groups | Density of FOXP3+ Tregs | Density of IDO+ DCs | CD11c+ DCs density | CD103+ DCs density | Langerin (CD207+ DCs density | EV+ cell density | Anti EV IgA level | Anti EV IgG level |
| sTNFRII | Controls  CD  CD+T1D  All CD cases |  | R=0.42 p=0.01 | R=0.37 p=0.04 |  |  | R=0.45 p=0.02 |  |  |
| GM-CSF | Controls  CD  CD+T1D  All CD cases |  | R=0.54 p=0.036 | R=-0.36 p=0.03 |  |  | R=0.91 p=0.006 |  | R=0.61 p=0.006 |
| Adiponectin | Controls  CD  CD+T1D  All CD cases |  |  |  |  |  | R=-0.63 p=0.01  R=-0.53 p=0.009 | R=0.58 p=0.03  R=0.64 p=0.007 |  |
| Leptin | Controls  CD  CD+T1D  All CD cases |  |  |  | R=0.38 p=0.02 |  | R=0.58 p=0.02  R=0.45 p=0.02 |  |  |
| Resistin | Controls  CD  CD+T1D  All CD cases |  |  |  |  |  |  |  |  |

*Only significant correlations between level of the studied cytokines and markers for Tregs, DCs, EV are presented.

Blank lines denote non significant correlations (p>0.05).
